# Impaired Structural Network Properties Caused by White Matter Hyperintensity Related to Cognitive Decline

**DOI:** 10.3389/fneur.2020.00250

**Published:** 2020-04-21

**Authors:** Dan Yang, Lili Huang, Caimei Luo, Mengchun Li, Ruomeng Qin, Junyi Ma, Pengfei Shao, Hengheng Xu, Bing Zhang, Yun Xu, Meijuan Zhang

**Affiliations:** ^1^Department of Neurology, Drum Tower Hospital, Medical School and The State Key Laboratory of Pharmaceutical Biotechnology, Institute of Brain Science, Nanjing University, Nanjing, China; ^2^Jiangsu Key Laboratory for Molecular Medicine, Medical School of Nanjing University, Nanjing, China; ^3^Jiangsu Province Stroke Center for Diagnosis and Therapy, Nanjing, China; ^4^Nanjing Neuropsychiatry Clinic Medical Center, Nanjing, China; ^5^Department of Radiology, Affiliated Drum Tower Hospital of Nanjing University Medical School, Nanjing, China

**Keywords:** white matter hyperintensity, cognitive impairment, white matter network, graph theoretical analysis, diffusion tensor imaging (DTI)

## Abstract

**Purpose:** There is a high correlation between white matter hyperintensity (WMH) and cognitive impairment (CI) in elderly people. However, not all WMH will develop into CI, and the potential mechanism of WMH-related CI is still unclear. This study aimed to investigate the topological properties of white matter structural network in WMH-related CI.

**Methods:** Forty-one WMH subjects with CI (WMH-CI), 42 WMH subjects without CI (WMH-no-CI), and 52 elderly healthy controls (HC) were recruited. Diffusion tensor imaging (DTI) fiber tractography and graph theoretical analysis were applied to construct the structural network. We compared network properties and clinical features among the three groups. Multiple linear regression analysis was performed to investigate the relationships among WMH volumes, impaired network properties, and cognitive functions in the WMH-CI group.

**Results:** Compared with the controls, both WMH groups showed decreased network strength, global efficiency, and increased characteristic path length (Lp) at the level of the whole brain. The WMH-CI group displayed more profound impairments of nodal efficiency and nodal path length (NLp) within multiple regions including precentral, cingulate, and medial temporal gyrus. The disrupted network properties were associated with CI and WMH burdens in the WMH-CI group. Furthermore, a mediation effect of NLp in the left inferior frontal gyrus was observed for the association between periventricular WMH (PWMH) and memory deficit.

**Conclusions:** Brain structural network in WMH-CI is significantly disturbed, and this disturbance is related to the severity of WMH and CI. Increased NLp in the left opercular part of inferior frontal gyrus (IFGoperc.L) was shown to be a mediation framework between PWMH and WMH-related memory, which shed light on investigating the underlying mechanisms of CI caused by WMH.

## Introduction

White matter hyperintensity (WMH), presented as high signal lesions on T2-weighted or fluid-attenuated inversion recovery (FLAIR) sequence, is frequently seen in elderly individuals (1). It is reported that WMH can be observed in 72–96% individuals over 60 years old (2). Additionally, the morbidities of periventricular white matter hyperintensity (PWMH) and deep white matter hyperintensity (DWMH) increase by 0.2 and 0.4%, respectively, with each additional year of age (3). According to Fazekas visual rating scales, WMH can be classified into four grades (Grade 0, no WMH; Grade 1, focal or punctate lesions; Grade 2, beginning confluent lesions; Grade 3, confluent lesions), among which the WMH of Grades 2–3 can cause disconnection syndromes (4).

Increasing evidences have confirmed that WMH can lead to cognitive impairment (CI) and is associated with an increased prevalence of stroke, dementia, and death (5, 6). Additionally, different lesion sites of WMH are closely related to different cognitive aspects. Compared with DWMH, PWMH is more likely leading to decreased information processing speed and memory deficit, while patients with DWMH have worse visuospatial function than patients with PWMH (7–9). However, not all subjects who displayed WMH will develop into CI. Therefore, the exact correlations between CI and WMH remain to be elucidated. One plausible rationale is that WMH burden can result in cognitive decline through making the previously connected cortex atrophy (10–12). Another possible mechanism is that WMH can disrupt white matter fibers and connectivity which play crucial roles in the information transportation of cortical–cortical or cortical–subcortical regions independent of cortical atrophy (13, 14). All these rationales were proposed based on studies of cerebral small vessel disease (CSVD) cohort (15, 16). However, the effects of WMH on CI in WMH cohort are largely unknown. One recent study has shown that lower WMH-related white matter connectivity was associated with worse cognitive function in the WMH population. But this study did not further explore the topological organization and network properties in these WMH subjects (17). Therefore, in this current study, we are going to evaluate the effects of WMH on the network topological architectures in WMH populations according to recruited elderly healthy controls (HC), WMH subjects without CI (WMH-no-CI), and WMH subjects with CI (WMH-CI).

Diffusion tensor imaging (DTI) is a powerful non-invasive imaging technique that can be used to trace white matter microstructures and abnormal white matter connectivity *in vivo* (18). Graph theoretical analysis is applied to construct a white matter structural network and provide information on the amount of integrations among brain regions. Nowadays, DTI and graph theoretical analysis have been increasingly applied to explore white matter integrity and structural network topological organization in multiple neurological diseases, including Alzheimer's disease (AD) (19), multiple sclerosis (20), amyotrophic lateral sclerosis (21), and schizophrenia (22). Furthermore, evidence of structural network changes and clinical relevance has already been presented in previous, larger and longitudinal, population-based studies (23–25).

Therefore, in the present study, we applied DTI tractography and graph analysis to elderly healthy, WMH-CI, and WMH-no-CI populations. We investigated the relationships among WMH burdens, white matter network properties, and CI. We hypothesized that WMH, one of the most common imaging manifestations observed in old population, can disrupt white matter network properties, which would be associated with CI.

## Materials and Methods

### Participants

The present research was carried out in accordance with the latest version of the Declaration of Helsinki and approved by the Nanjing Drum Tower Hospital Research Ethics Committee. Overall, 83 subjects with WMH (Fazekas scale 2 or 3) and 52 healthy elderly controls were recruited from January 2017 to April 2019 in inpatients and outpatients of the neurological department, Nanjing Drum Tower Hospital. WMH was diagnosed independently and unanimously by two radiologists, who visually evaluated MRI without knowledge of the participants' clinical profiles. WMH subjects were further divided into WMH-CI group (*n* = 41) and WMH-no-CI group (*n* = 42) based on the Beijing version of the Montreal Cognitive Assessment (MoCA-BJ). All participants were provided written informed consents and underwent multimodal MRI scans and standardized diagnostic evaluations, including demographic data, vascular risk factors, and an examination of neuropsychological status.

The inclusion criteria for WMH subjects were as follows: (1) age between 50 and 80 years; (2) presence of grade 2 or 3 WMH according to Fazekas scale on FLAIR; (3) no contraindications to MRI. Exclusion criteria were as follows: (1) a history of ischemic stroke with infarct size more than 1.5 cm in diameter or cardiogenic cerebral embolism; (2) cerebral hemorrhage; (3) internal carotid artery or vertebral artery stenosis (>50%) or coronary atherosclerosis heart disease; (4) WMH due to immune-mediated inflammatory demyelinating disease (multiple sclerosis, neuromyelitis optica, acute disseminated encephalomyelitis), metabolic leukodystrophy and genetic leukoencephalopathy; (5) other neurological disorders, such as AD, Parkinson's disease, epilepsy; (6) systemic disease, such as cancer, shock, and anemia; (7) prominent impairments of audition or vision.

### Neuropsychological Examination

Each subject underwent a standardized neuropsychological test protocol, including the mental status, global cognitive assessments, and multiple cognitive domain examinations. Hamilton Depression Rating Scale (HAMD) and Hamilton Anxiety Rating Scale (HAMA) were used to test the mental status of all subjects. Global cognitive function was evaluated by Mini Mental State Examination (MMSE) and MoCA-BJ. WMH subjects with MoCA-BJ scores lower than education-adjusted norms (the cut-off was ≤ 19 for 1–6 years of education, ≤ 24 for 7–12 years of education and <26 for >12 years of education) were defined as the WMH-CI group (*n* = 41) and other WMH subjects were defined as the WMH-no-CI group (*n* = 42). The raw test scores were converted to Z-scores which calculate the compound cognitive index. Executive function is a compound score of the average Z-scores of Trail Making Test-B (TMT-B) and Stroop Color and Word Test-C (Stroop C). Information processing speed was calculated as the average Z-scores of Trail Making Test-A (TMT-A), Stroop Color and Word Tests A and B (Stroop A and B). Memory was calculated as the mean of the Z-scores of Wechsler Memory Scale-Visual Reproduction-Delayed Recall (VR-DR) and Auditory Verbal Learning Test-Long Delayed Recall (AVLT-DDR) representing visual memory and verbal memory, respectively. Visuospatial processing function was a compound score that included the mean of the Z-scores of Clock Drawing Test (CDT) and Visual Reproduction-Copy (VRC). Language function was consisted of Category Verbal Fluency (CVF) and Boston Naming Test (BNT).

### MRI Scanning

All of the participants were examined on a Philips 3.0-T scanner (Philips Medical Systems, Netherlands). The examination protocol included the high-resolution T1-weighted turbo gradient echo sequence [repetition time (TR) = 9.8 ms, flip angle (FA) = 8°, echo time (TE) = 4.6 ms, field of view (FOV) = 250 mm × 250 mm, number of slices = 192, acquisition matrix = 256 × 256, thickness = 1.0 mm], the FLAIR sequence [TR = 4,500 ms, TE = 333 ms, time interval (TI) = 1,600 ms, number of slices = 200, voxel size = 0.95 mm × 0.95 mm × 0.95 mm, acquisition matrix = 270 × 260] and the gradient-recalled echo planar imaging (EPI) sequence [TR = 2,000 ms, FA = 90°, TE = 30 ms, number of slices = 35, acquisition matrix = 64 × 64, FOV = 240 mm × 240 mm, thickness = 4.0 mm]. DTI data were obtained using an EPI sequence with the following parameters: in 32 non-collinear directions diffusion encoding (b = 1,000 s/mm^2^ for each direction) and one image with no diffusion weighting (b = 0 s/mm^2^), TR = 9,154 ms, TE = 55 ms, FA = 90°, matrix size = 112 × 112, FOV = 224 mm × 224 mm, slice thickness = 2.5 mm. The total scan takes 13 min and 10 s. Additionally, axial T_2_-weighted, diffusion weighted imaging (DWI) sequence, and susceptibility weighted imaging were collected to detect acute or subacute infarctions, cerebral microbleeds. Wisconsin White Matter Hyperintensity Segmentation Toolbox **(**https://sourceforge.net/projects/w2mhs) was used to semiautomatically quantify total white matter hyperintensity (TWMH) volume including DWMH and PWMH based on T1-weighted and FLAIR images. The total brain volume (TBV), gray matter volume, and white matter volume were automatically obtained using Statistical Parametric Mapping (SPM8, http://www.fil.ion.ucl.ac.uk/spm).

### Diffusion Tensor Imaging Processing and Network Reconstruction

The processing of DTI data and network reconstruction were carried out by PANDA software, and its default pipeline setting (http://www.nitrc.org/projects/panda/) (26) contains processing functions from FSL (https://fsl.fmrib.ox.ac.uk/fsl/fslwiki), DiffusionToolkit (http://www.trackvis.org/dtk/), and MRIcron (https://www.nitrc.org/projects/mricron). The preprocessing of DTI data included the following steps: (1) converting DICOM files into NIFTI images, (2) estimating the brain mask, (3) cropping the raw images, (4) correcting for the distortion and head motion by registering the diffusion-weighted images to the b0 images with an affine transformation, and (5) calculating the DTI metrics. Network nodes were defined as 90 brain regions segmented by the automated anatomical labeling (AAL) template (27). Skull-stripped T1-weighted images were non-linearly registered to its corresponding FA native diffusion space using an affine transformation and then normalized to the Montreal Neurological Institute (MNI) 152 template using Functional MRI of the Brain Non-linear Image Registration Tool (FNIRT), part of the FSL tools. Whole-brain deterministic tractography was performed to define network edges. White matter tracts were reconstructed for each individual dataset using the Fiber Assignment by Continuous Tracking (FACT) algorithm embedded in the Diffusion Toolkit. Fiber tracking was terminated when the tracking streamline encountered voxels with fractional anisotropy (FA) <0.2 or the turning angle exceeded 45°. For every pair of brain nodes/regions defined above, fibers with two end points located in their respective masks were considered as the network edges linking the two nodes. Based on the linking fibers, PANDA calculated three basic weighted matrices: number-weighted matrix (M^FN^), FA-weighted matrix (M^FA^), and length-weighted matrix (M^L^). The values of the elements M(i, j)^FN^, M(i, j)^FA^, and M(i, j)^L^ represent the fiber numbers (FN), averaged FA, and averaged length of linking fibers between node i and node j, respectively. The obtained FN was binarized after determining a threshold of FN >3 to reduce the false-positive connectivity that resulted from noise. According to previous studies (28, 29), we used the M^FN^ and M^FA^ to define the weight edge, i.e., M(i,j)^FA.FN^ = M(i, j)^FN^ × M(i, j)^FA^, that is, the value of multiplying FN by the averaged FA along the fiber bundles connected to a pair of cortical regions (node i and node j) was used to weight the edge.

### Graph Theoretical Analysis

Using the Gretna Toolbox (http://www.nitrc.org/projects/gretna/) based on Brain Connectivity Toolbox, we calculated the network density defined as the total number of edges in a network divided by the possible number of edges, and average network strength defined as the mean sum of all weighted edges for every node. To further investigate the topological organization of the network, we calculated global and nodal clustering coefficient (Cp), characteristic path length (Lp), global efficiency (Eg), and local efficiency (Eloc), respectively. The detailed definitions, calculating formula, and descriptions of these topological properties for a network G with N nodes and V edges are as follows (30):

### Global Topological Properties

Cp at the level of network indicates the extent of local cliquishness or interconnectivity of a network, which can be calculated as:

(1) $$\begin{array}{r} \text{Cp}\left(\text{G}\right)=\frac{1}{N}\underset{i\in N}{\sum }\frac{\sum_{j,k\in N}{\left(W_{ij}W_{ik}W_{jk}\right)}^{\frac{1}{3}}}{K_{i}\left(K_{i}-1\right)} \end{array}$$

*K*_i_ is the degree of node *i*, and *W*_ij_ is the weight between node *i* and *j* in the network.

Characteristic Lp at the level of network is an indicator of overall network connectedness and quantifies the parallel information propagation ability. It is calculated as:

(2) $$\begin{array}{r} \text{Lp}\left(\text{G}\right)=\frac{1}{1/\left(N\left(N-1\right)\right)\sum_{i=1}^{N}\sum_{j\neq i}^{N}1/L_{ij}} \end{array}$$

*L*_ij_ is the characteristic Lp between nodes *i* and *j*.

Eg is defined as the inverse of the harmonic mean of shortest Lp between each pair of nodes within the network. It measures efficiently the information communication capacity through the whole network and is calculated as:

(3) $$\begin{array}{r} \text{Eg}\left(\text{G}\right)=\frac{1}{N\left(N-1\right)}\underset{i\neq j\in G}{\sum }\frac{1}{dij} \end{array}$$

*d*_*ij*_ is the shortest Lp between node i and j in the network.

Eloc at the level of network reveals how efficiently the information is communicated among the neighbors of a given node when that node is removed, showing how fault tolerant the network is. It is calculated as:

(4) $$\begin{array}{r} \text{Eloc}\left(\text{G}\right)=\frac{1}{N}\underset{i\in N}{\sum }\left(\frac{1}{N_{G_{i}}\left(N_{G_{i}}-1\right)}\underset{j\neq k\in v}{\sum }\frac{1}{L_{jk}}\right) \end{array}$$

*G*_i_ is the subgraph composed of the nearest neighbors of node *i*. *L*_jk_ is the shortest Lp between node *j* and node *k* of subgraph about *i*.

### Nodal Topological Properties

Nodal Cp measures the likelihood of neighbor node connected to each other, which can be calculated as:

(5) $$\begin{array}{r} \text{Cp}\left(\text{i}\right)=\frac{Ei}{\frac{1}{2}ki\left(ki-1\right)} \end{array}$$

*Ei* is the actual number of edges between neighbor nodes connected to node i.

Nodal Lp (Nlp) quantifies the mean distance or routing efficiency between one node and all the other nodes in the network. It is calculated as:

(6) $$\begin{array}{r} \text{Lp}\left(\text{i}\right)=\frac{1}{N-1}\underset{i\neq j\notin G}{\sum }dij \end{array}$$

*d*_*ij*_ is the shortest Lp between node i and j in the network.

Nodal Eg characterizes the efficiency of parallel information transfer of one node in the network, which can be calculated as:

(7) $$\begin{array}{r} \text{Eg}\left(\text{i}\right)=\frac{1}{N-1}\underset{i\neq j\in G}{\sum }\frac{1}{dij} \end{array}$$

*d*_*ij*_ is the shortest Lp between node i and j in the network.

Nodal Eloc measures how efficient the communication is among the first neighbors of one node when it is removed, which can be calculated as:

(8) $$\begin{array}{r} \text{Eloc}\left(\text{i}\right)=\underset{i\in G}{\sum }Eg\left(Gi\right) \end{array}$$

Gi is the subgraph consisting of node i and its local neighbors.

### Statistical Analysis

WMH volumes were log-transformed to obtain a normal distribution. Differences in demographic, clinical, volume, and neuropsychological data across the three groups were analyzed using one-way analysis of variance (ANOVA), chi-squared (**χ**^**2**^) test, or Kruskal–Wallis test in case of non-normality which was performed in the SPSS 22.0 software (IBM Corp., Armonk, NY). *P* < 0.05 was considered statistically significant.

Group differences in the structural network (Cp, characteristic Lp, Eg, and Eloc) at the level of network were explored using one-way analysis of covariance (ANCOVA), adjusted for age, sex, education years (with Bonferroni-corrected *post hoc t*-test, *P* = 0.05/3). Then, group differences in nodal efficiency and NLp were examined with ANCOVA adjusted for age, sex, education years with false discovery rate (FDR) correction (*q* = 0.01) for multiple comparisons. Subsequently, for the FDR-corrected statistically significant brain regions, a *post hoc* analysis was performed to investigate group differences between any two groups, additionally correcting for multiple comparisons with Bonferroni correction which is standard in SPSS 22.0 when covariates are entered into the model. Then, multiple linear regression analysis was performed to investigate relationships among log-transformed WMH volume, white matter network properties, and cognitive function in WMH-CI group adjusting for age, gender, and education years.

Additionally, mediation analysis was performed to explore whether network properties were involved in the relationship between WMH volumes and cognitive function, adjusting for age, gender, and education years. The primary estimates of interest were the degree of the changes in the direct path between WMH volume and cognition, labeled c in the bivariate models and c' in the full mediating models, and the indirect path from WMH volume to cognition through the white matter network metric: the product of paths a and b. We computed the bias-corrected 95% confidence intervals for the size of the mediating effects with bootstrapping (k = 5,000 samples). The mediating effect is said to be present if the 95% confidence interval does not contain zero. Mediation analyses were conducted in PROCESS for the SPSS 22.0 framework.

## Results

### Demographic, Clinical, and Neuropsychological Data

Demographic, clinical, and neuropsychological characteristics for the three groups were shown in Table 1. There were no differences in gender, education years, vascular risk factors, the number of lacunars and cerebral microbleeds, and HAMD and HAMA scores among the three groups (all *P* > 0.05) except for ages, cognitive functions, and WMH volumes (*P* < 0.05). We hereby removed age effect in all the following network analyses. In contrast with subjects in the WMH-no-CI and HC groups, WMH-CI subjects exhibited poorer cognitive performance on the MMSE (*P* < 0.001) and MoCA-BJ (*P* < 0.001) tests. Additionally, WMH-CI subjects showed worse executive function (*P* < 0.001), information processing speed (*P* < 0.001), language function (*P* < 0.003), memory (*P* < 0.001), and visuospatial processing function (*P* = 0.002) than the other two groups (Table 1). As shown in Table 1, both of the WMH groups displayed significantly larger TWMH, PWMH, and DWMH volume than those of the HC group (all *P* < 0.05). However, WMH volume was similar between the WMH-CI group and the WMH-no-CI group (*P* > 0.05). Furthermore, no significant differences in TBV, gray matter volume, and white matter volume were found among the three groups (*P* > 0.05).

**Table 1:** Demographic, clinical, volume, and neuropsychological data.

**Item**	**HC (*n* = 52)**	**WMH-no-CI (*n* = 42)**	**WMH-CI (*n* = 41)**	**F/x^**2**^**	***P*-value**
**Demographics**					
Age (years)	63.19 ± 6.59	66.43 ± 6.94	67.81 ± 6.76^a^	5.81	**0.004**
Gender, male (% male)	25 (48.08)	15 (35.71)	19 (46.34)	1.61	**0.447**
Education (years)	12 (3,19)	11.5 (2,19)	9 (2.5,16)	3.18	**0.204**
**Vascular risk factors**					
Hypertension, *n* (%)	31 (59.62)	27 (64.29)	33 (80.49)	4.82	0.09
Diabetes, *n* (%)	9 (17.31)	10 (23.81)	8 (19.51)	0.62	0.732
hyperlipidemia, *n* (%)	7 (13.46)	8 (19.04)	9 (21.95)	1.06	0.589
Smoking, *n* (%)	8 (15.38)	7 (16.67)	8 (19.51)	0.28	0.868
Lacunars, *n* (%)	3 (5.77)	5 (11.90)	4 (9.76)	1.14	0.567
microbleeds, *n* (%)	8 (15.38)	13 (30.95)	11 (26.83)	3.43	0.18
NO. Lacunars	0 (0,2)	0 (0,2)	0 (0,3)	3.3	0.192
NO. microbleeds	0 (0,2)	0 (0,3)	0 (0,5)	1.84	0.396
**Volume data**					
TBV (ml)	1277.75 ± 165.05	1279.43 ± 142.29	1314.28 ± 162.72	0.73	0.481
TWMH volume (ml)	1.66 ± 0.82	6.08 ± 2.33^a^	7.46 ± 5.33^a^	93.88	** <0.001**
PWMH volume (ml)	1.29 ± 0.53	4.79 ± 1.75^a^	5.48 ± 2.15^a^	58.98	** <0.001**
DWMH volume (ml)	0.37 ± 0.38	1.29 ± 0.88^a^	1.98 ± 2.15^a^	91	** <0.001**
Gray matter volume (ml)	519.25 ± 72.73	518.96 ± 63.77	527.32 ± 66.79	0.203	0.817
White matter volume (ml)	460.11 ± 69.42	443.93 ± 61.43	452.82 ± 72.20	0.624	0.538
**Neuropsychological data**					
Mental status
HAMD	5.77 ± 4.59	4.95 ± 4.62	6.78 ± 5.04	3.89	0.143
HAMA	8.288 ± 6.48	8.12 ± 6.92	9.63 ± 6.67	1.41	0.494
General cognitive function	0.40 ± 0.56	0.32 ± 0.51	−0.84 ± 1.04^a^^b^	38.86	** <0.001**
MMSE	0.31 ± 0.64	0.18 ± 0.60	−0.58 ± 1.40^a^^,^ ^b^	11.57	** <0.001**
MoCA-BJ	0.49 ± 0.64	0.46 ± 0.60	−1.09 ± 0.84^a^^,^ ^b^	72.88	** <0.001**
Executive Function	0.30 ± 0.51	0.68 ± 0.77	−0.45 ± 0.83^a^^,^ ^b^	13.28	** <0.001**
TMT-B (minus value)	0.35 ± 0.83	0.10 ± 0.91	−0.54 ± 0.07^a^^,^ ^b^	10.56	** <0.001**
Stroop C (minus value)	0.25 ± 0.60	0.04 ± 1.02	−0.36 ± 1.27^a^	4.56	**0.012**
Information processing speed	0.28 ± 0.10	0.12 ± 0.11	−0.48 ± 0.11^a^^,^ ^b^	18.34	** <0.001**
TMT-A (minus value)	0.36 ± 0.62	0.05 ± 0.88	−0.51 ± 1.28^a^^,^ ^b^	9.71	** <0.001**
Stroop A (minus value)	0.39 ± 0.56	0.09 ± 0.83	−0.58 ± 1.30^a^^,^ ^b^	12.94	** <0.001**
Stroop B (minus value)	0.28 ± 0.79	0.23 ± 0.79	−0.59 ± 1.19^a^^b^	11.94	** <0.001**
Language function	0.15 ± 0.92	0.17 ± 0.82	−0.36 ± 0.61^b^	5.91	**0.003**
CVF	0.08 ± 0.92	0.14 ± 1.34	−0.24 ± 0.60	1.82	0.167
BNT	0.22 ± 1.18	0.20 ± 0.66	−0.47 ± 0.90^a^^,^ ^b^	7.24	**0.001·**
Memory	0.22 ± 0.69	0.22 ± 0.78	−0.51 ± 0.74^a^^,^ ^b^	14.11	** <0.001**
AVLT-DDR	0.29 ± 0.86	0.25 ± 1.03	−0.62 ± 0.87^a^^,^ ^b^	13.42	** <0.001**
VR-DR	0.16 ± 0.10	0.19 ± 0.98	−0.40 ± 0.93^a^^,^ ^b^	4.9	**0.009**
Visuospatial processing function	0.14 ± 0.63	0.16 ± 0.59	−0.34 ± 0.96^a^^,^ ^b^	6.3	**0.002**
VR-C	0.13 ± 0.68	0.14 ± 0.75	−0.30 ± 1.43	2.72	0.07
CDT	0.15 ± 0.93	0.18 ± 0.80	−0.38 ± 0.18^a^^,^ ^b^	4.41	**0.014**

*Values were presented as mean ± SD, median with minimum and maximum or absolute numbers with percentages. P-values (<0.05) appeared in bold*,

a *P <0.05 vs. controls*.

b *P <0.05 vs*.

*WMH-no-CI group. AVLT-DDR, Auditory Verbal Learning Test-long delayed recall; BNT, Boston Naming Test; CI, cognitive impairment; CDT, Clock Drawing Test; CVF, Category language fluency; DWMH, deep white matter hyperintensities; HAMA, Hamilton anxiety rating Scale; HAMD, Hamilton depression rating scale; HC, healthy controls; MMSE, Mini-Mental State Examination; MoCA-BJ, Beijing version of the Montreal Cognitive Assessment; PWMH, periventricular white matter hyperintensities; Stroop A, Stroop Color and Word Tests A; Stroop B, Stroop Color and Word Tests B; Stroop C, Stroop Color and Word Tests C; TBV, total brain volume; TMT-A, Trail Making Test-A; TMT-B, Trail Making Test-B; TWMH, total white matter hyperintensity; VR-C, visual reproduction-copy; VR-DR, visual reproduction-delay recall; WMH, white matter hyperintensities*.

### Group Differences in Global Network Properties

**Figure 1:**
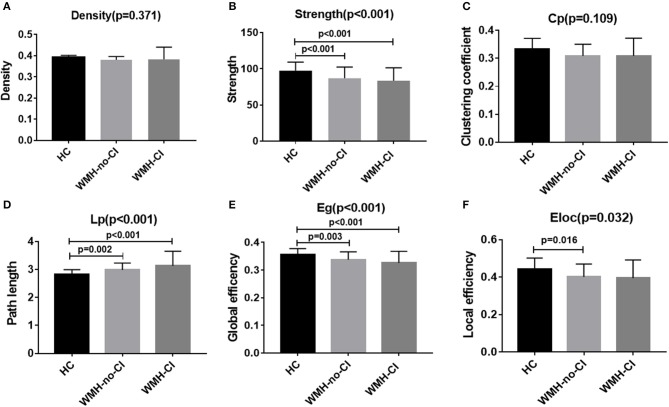
Bar graph of group differences on the whole-brain network properties. Data were presented as mean ± SD. **(A,C)** No significant difference in network density and clustering coefficient among the three groups (ANCOVA, *P* > 0.05). **(B,E)** WMH-no-CI and WMH-CI groups displayed lower network strength and global efficiency than HC group (ANCOVA, *P* < 0.001; Bonferroni-corrected, *P* < 0.05/3). **(D)** WMH-no-CI and WMH-CI groups displayed higher path length than HC group (ANCOVA, *P* < 0.001; Bonferroni-corrected, *P* < 0.05/3). **(F)** WMH-no-CI group displayed lower local efficiency than HC group (ANCOVA, *P* < 0.05; Bonferroni-corrected, *P* < 0.05/3). Cp, clustering coefficient; CI, cognitive impairment; Eg, global efficiency; Eloc, local efficiency; HC, healthy controls; Lp, path length; WMH, white matter hyperintensity.

Global network analysis was shown in Figure 1 and Table S1. No significant differences were detected among the three groups in network density (*P* = 0.371; Figure 1A) and Cp (*P* = 0.109; Figure 1C). Both WMH-CI and WMH-no-CI groups displayed decreased network strength (*P* < 0.001 in WMH-CI group, *P* < 0.001 in WMH-no-CI group vs. HC group, Bonferroni-corrected, *P* < 0.05/3), decreased Eg (*P* < 0.001 in WMH-CI group, *P* = 0.003 in WMH-no-CI group vs. HC group, Bonferroni-corrected, *P* < 0.05/3), and increased characteristic Lp (*P* < 0.001 in WMH-CI group, *P* = 0.002 in WMH-no-CI group vs. HC group, Bonferroni-corrected, *P* < 0.05/3) in comparison with the HC group (Figures 1B,D,E). In addition, subjects of WMH-no-CI showed lower Eloc than controls (*P* = 0.010, Bonferroni-corrected, *P* < 0.05/3) (Figure 1F). Relative to the WMH-no-CI group, the WMH-CI group showed lower network strength, lower Eg, and higher characteristic Lp. However, it did not achieve statistical significance.

### Group Differences in Nodal Network Properties

**Figure 2:**
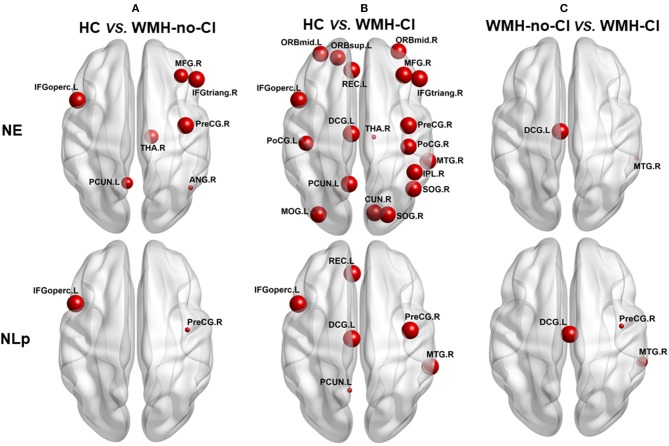
*Post hoc* analyses of group differences in nodal efficiency and nodal path length. The impaired regions were marked with red nodes. Node size represented the between-group differences (*P* < 0.05, Bonferroni-corrected). **(A)** *post hoc* analyses of group deferences in nodal efficiency and nodal path length between HC and WNH-no-CI. **(B)** *post hoc* analyses of group deferences in nodal efficiency and nodal path length between HC and WNH-CI. **(C)** *post hoc* analyses of group deferences in nodal efficiency and nodal path length between WNH-no-CI and WMH-CI. ANG, angular gyrus; CI, cognitive impairment; CUN, cuneus gyrus; DCG, median cingulate and paracingulate gyrus; HC, health control; IFGoperc, opercular part of inferior frontal gyrus; IFGtriang, triangular part of inferior frontal gyrus; IPL, inferior parietal; L, left; MFG, middle frontal gyrus; MOG, middle occipital gyrus; MTG, middle temporal gyrus; NE, nodal efficiency; NLp, nodal path length; ORBmid, orbital part of middle frontal gyrus; ORBmid, orbital part of middle frontal gyrus; ORBsup, orbital part of superior frontal gyrus; PCUN, precuneus; PoCG, postcentral gyrus; PoCG, postcentral gyrus; PreCG, precentral gyrus; REC, rectus gyrus; R, right; SOG, superior occipital gyrus; THA, thalamus; WMH, white matter hyperintensity.

Nodal network analysis was shown in Figure 2 and Table S2. Among the three groups, we found significant differences of Lp in six brain regions and Eg in 19 brain regions out of the 90 brain regions (Table S2). *Post-hoc* analysis showed that nodal properties were widely altered in WMH-CI and WMH-no-CI groups in contrast with those in the HC group (25 nodes in WMH-CI vs. HC and nine nodes in WMH-no-CI vs. HC, *P* < 0.05, Bonferroni-corrected) (Figures 2 A,B). To reveal nodes specifically related to CI, we carried out a *post hoc* comparison between WMH-CI and WMH-no-CI. In contrast with WMH-no-CI group, WMH-CI subjects displayed longer Lp in three nodes [right precentral gyrus (PreCG.R); left median cingulate and para cingulate gyri (DCG.L); right middle temporal gyrus (MTG.R)] and lower Eg in two nodes (DCG.L; MTG.R; *P* < 0.05, Bonferroni-corrected, Figure 2C).

### Multiple Linear Regressions of White Matter Hyperintensity Volume, White Matter Network Metrics, and Cognitive Function in WMH-CI Subjects

Multiple linear regression analysis was used to explore the relationship among WMH volume, network properties, and cognition function adjusted for age, gender, and education years in subjects of WMH-CI. Firstly, we found that TWMH and PWMH were negatively associated with CI in multiple domains including general cognitive function, executive function, information processing speed, language function, memory, and visuospatial processing function. However, DWMH was just negatively associated with memory and visuospatial processing function (all *P* < 0.05; Table 2). Secondly, we found TWMH, PWMH, and DWMH were all positively associated with characteristic Lp and negatively associated with Eg at the level of global network. When analyzing the relationships between WMH and nodal network properties, we found that TWMH and DWMH were positively associated with NLp of PreCG.R, left opercular part of inferior frontal gyrus (IFGoperc.L), left precuneus (PCUN.L), and MTG.R. PWMH was positively associated with NLp of PreCG.R, IFGoperc.L, and MTG.R. On the other hand, TWMH and PWMH were negatively associated with nodal efficiency of PreCG.R, right middle frontal gyrus (MFG.R), right triangular part of inferior frontal gyrus (IFGtriang.R), right cuneus (CUN.R), right superior occipital gyrus (SOG.R), left middle occipital gyrus (MOG.L), and left postcentral gyrus (PoCG.L). DWMH was negatively associated with nodal efficiency of MFG.R, IFGtriang.R, SOG.R, MOG.L, PoCG.L, and MTG.R (all *P* < 0.05; Table 3). Thirdly, as shown in Table 4, disrupted white matter network properties at the level of network were associated with CI in multiple domains. Negative association was observed between characteristic Lp at the level of global network and general cognitive ability, information processing speed, language function, and visuospatial processing function (all *P* < 0.05). Similarly, positive association was observed between Eg and information processing speed, language function, visuospatial processing function (all *P* < 0.05). Additionally, we further analyzed the associations between nodal network metrics and cognitive domains. As shown in Table 4, we found that higher NLp of PreCG.R and MTG.R and lower nodal efficiency of MFG.R, SOG.R, MOG.L, right inferior parietal (IPL.R), and right angular gyrus (ANG.R) were associated with worse general cognitive ability (all *P* < 0.05). Higher NLp of PCUN.L and lower nodal efficiency of PreCG.R, IFGtriang.R, CUN.R, SOG.R, PoCG.L, PCUN.L, and right thalamus (THA.R) were associated with worse executive function (all *P* < 0.05). Higher NLp of PreCG.R, IFGoperc.L, left median cingulate and paracingulate gyri (DCG.L), PCUN.L, and MTG.R and lower nodal efficiency of PreCG.R, MFG.R, CUN.R, SOG.R, MOG.L, IPL.R, ANG.R, and MTG.R were associated with worse information processing speed (all *P* < 0.05). Higher NLp of PreCG.R, DCG.L, and MTG.R, and lower nodal efficiency of PreCG.R, MFG.R, right medial orbitofrontal gyrus (ORBmid.R), IFGtriang.R, DCG.L, CUN.R, SOG.R, MOG.L, PoCG.R, and MTG.R were associated with worse language function (all *P* < 0.05). Higher NLp of PreCG.R, IFGoperc.L, DCG.L, PCUN.L, and MTG.R, and lower nodal efficiency of PreCG.R, MFG.R, ORBmid.R, SOG.R, MOG.L, PoCG.L, IPL.R, ANG.R, and MTG.R were associated with visuospatial processing function (all *P* < 0.05). Higher NLp of IFGoperc.L and lower nodal efficiency of IFGoperc.L, IFGtriang.R, PoCG.L, and ANG.R were associated with worse memory (all *P* < 0.05).

**Table 2:** Multiple linear regression analyses of WMH volumes and cognitive function in WMH-CI.

**Items**	**Lg TWMH**		**Lg PWMH**		**Lg DWMH**	
	**Standardized coefficients β**	***P*-value**	**Standardized coefficients β**	***P*-value**	**Standardized coefficients β**	***P*-value**
General cognitive function	−0.317	**0.029**	−0.281	**0.042**	−0.186	0.217
Executive function	−0.445	**0.007**	−0.397	**0.011**	−0.306	0.075
Information processing speed	−0.384	**0.023**	−0.355	**0.027**	−0.198	0.261
Language function	−0.406	**0.007**	−0.413	**0.003**	−0.233	0.139
Memory	−0.415	**0.01**	−0.313	**0.042**	−0.453	**0.005**
Visuospatial processing function	−0.411	**0.012**	−0.329	**0.036**	−0.396	**0.017**

*P-values (<0.05) appeared in bold. DWMH, deep white matter hyperintensities; PWMH, periventricular white matter hyperintensities; TWMH, total white matter hyperintensities*.

**Table 3:** Multiple linear regression analyses of WMH volumes and cognitive function in WMH-CI.

**Dependent variable**	**Independent variable (Standardized Coefficients** **β**, ***P*****-value)**					
	**Lg TWMH**		**Lg PWMH**		**Lg DWMH**	
Eg	**−0.39**	**0.021**	**−0.343**	**0.032**	**−0.351**	**0.042**
Lp	**0.468**	**0.005**	**0.408**	**0.01**	**0.397**	**0.022**
PreCG.R_NE	**−0.391**	**0.017**	**−0.337**	**0.03**	−0.32	0.057
ORBsup.L_NE	−0.094	0.605	−0.101	0.556	−0.0819	0.658
MFG.R_NE	**−0.448**	**0.011**	**−0.351**	**0.037**	**−0.467**	**0.009**
ORBmid.L_NE	0.163	0.348	0.112	0.498	0.148	0.402
ORBmid.R_NE	−0.253	0.157	−0.18	0.289	−0.28	0.123
IFGoperc.L_NE	−0.337	0.053	−0.28	0.091	−0.349	0.05
IFGtriang.R_NE	**−0.519**	**0.003**	**−0.436**	**0.009**	**−0.5**	**0.005**
REC.L_NE	−0.14	0.437	−0.172	0.308	−0.096	0.66
DCG.L_NE	−0.261	0.13	−0.222	0.175	−0.247	0.16
CUN.R_NE	**−0.346**	**0.028**	**−0.341**	**0.021**	−0.182	0.269
SOG.R_NE	**−0.493**	**0.003**	**−0.44**	**0.006**	**−0.401**	**0.021**
MOG.L_NE	**−0.544**	**0.001**	**−0.495**	**0.002**	**−0.431**	**0.015**
PoCG.L_NE	−0.166	0.35	−0.092	0.583	−0.24	0.181
PoCG.R_NE	−0.163	0.322	−0.128	0.409	−0.165	0.324
IPL.R_NE	−0.203	0.233	−0.118	0.465	−0.315	0.066
ANG.R_NE	−0.296	0.099	−0.241	0.157	−0.326	0.073
PCUN.L_NE	−0.247	0.15	−0.178	0.273	−0.285	0.1
THA.R_NE	−0.171	0.336	−0.17	0.311	−0.129	0.478
MTG.R_NE	**−0.446**	**0.009**	**−0.366**	**0.024**	**−0.403**	**0.021**
PreCG.R_NLp	**0.494**	**0.002**	**0.426**	**0.005**	**0.392**	**0.018**
IFGoperc.L_NLp	**0.45**	**0.007**	**0.374**	**0.019**	**0.438**	**0.01**
REC.L_NLp	0.114	0.523	0.165	0.327	0.039	0.832
DCG.L_NLp	0.325	0.059	0.273	0.094	0.297	0.091
PCUN.L_NLp	**0.362**	**0.032**	0.28	0.082	**0.358**	**0.037**
MTG.R_NLp	**0.553**	**0.001**	**0.472**	**0.003**	**0.446**	**0.01**

*P-values (<0.05) appeared in bold. ANG, angular gyrus; CUN, cuneus gyrus; DCG, median cingulate and paracingulate gyrus; DWMH, deep white matter hyperintensities; Eg, global efficiency; IFGoperc, opercular part of inferior frontal gyrus; IFGtriang, triangular part of inferior frontal gyrus; IPL, inferior parietal; L, left; Lp; path length; MFG, middle frontal gyrus; MOG, middle occipital gyrus; MTG, middle temporal gyrus; NE, nodal efficiency; NLp, nodal path length; ORBmid, orbital part of middle frontal gyrus; ORBmid, orbital part of middle frontal gyrus; ORBsup, orbital part of superior frontal gyrus; PCUN, precuneus; PoCG, postcentral gyrus; PoCG, postcentral gyrus; PreCG, precentral gyrus; PWMH; periventricular white matter hyperintensities; REC, rectus gyrus; R, right; SOG, superior occipital gyrus; THA, thalamus; TWMH, total white matter hyperintensities*.

**Table 4:** Multiple linear regression analyses of network metrics and cognitive function in WMH–CI.

**Independent variable**	**Dependent variable (Standardized coefficients** **β**, ***P*****-value)**											
	**General cognitive ability**		**Executive function**		**Information processing speed**		**Language function**		**Visuospatial processing function**		**Memory**	
Eg	0.163	0.252	0.3	0.063	**0.342**	**0.036**	**0.355**	**0.016**	**0.421**	**0.007**	−0.279	0.074
Lp	**−0.302**	**0.028**	−0.284	0.078	**−0.438**	**0.006**	**−0.388**	**0.007**	**−0.457**	**0.003**	0.262	0.098
PreCG.R_NE	0.254	0.081	**0.331**	**0.046**	**0.407**	**0.014**	**0.374**	**0.012**	**0.439**	**0.006**	0.292	0.073
ORBsup.L_NE	0.07	0.615	−0.041	0.968	0.036	0.821	0.142	0.324	0.265	0.086	0.154	0.317
MFG.R_NE	**0.273**	**0.04**	0.112	0.475	**0.335**	**0.03**	**0.371**	**0.007**	**0.394**	**0.008**	0.18	0.236
ORBmid.L_NE	−0.072	0.619	−0.005	0.976	−0.021	0.898	0.105	0.485	0.176	0.287	0.075	0.64
ORBmid.R_NE	0.124	0.369	0.214	0.175	0.174	0.279	**0.283**	**0.046**	**0.309**	**0.044**	0.196	0.201
IFGoperc.L_NE	0.048	0.346	0.223	0.163	0.226	0.163	0.194	0.183	0.269	0.087	**0.359**	**0.018**
IFGtriang.R_NE	0.049	0.718	**0.486**	**0.001**	0.256	0.102	**0.283**	**0.042**	0.235	0.125	**0.343**	**0.02**
REC.L_NE	−0.124	0.373	−0.004	0.98	−0.08	0.625	−0.058	0.693	−0.107	0.501	0.189	0.222
DCG.L_NE	0.057	0.691	0.247	0.129	0.29	0.077	**0.335**	**0.021**	0.293	0.067	0.204	0.201
CUN.R_NE	0.196	0.201	**0.341**	**0.049**	**0.473**	**0.006**	**0.323**	**0.04**	0.294	0.089	0.12	0.487
SOG.R_NE	**0.287**	**0.037**	**0.343**	**0.03**	**0.47**	**0.002**	**0.309**	**0.032**	**0.331**	**0.034**	0.291	0.06
MOG.L_NE	**0.438**	**0.001**	0.218	0.169	**0.534**	** <0.001**	**0.381**	**0.006**	**0.455**	**0.002**	0.261	0.088
PoCG.L_NE	0.105	0.453	**0.458**	**0.003**	0.256	0.114	0.207	0.156	**0.307**	**0.049**	**0.378**	**0.012**
PoCG.R_NE	0.069	0.648	0.156	0.371	0.155	0.38	**0.331**	**0.033**	0.141	0.415	0.076	0.45
IPL.R_NE	**0.31**	**0.029**	0.026	0.876	**0.462**	**0.004**	0.142	0.352	**0.359**	**0.026**	0.266	0.099
ANG.R_NE	**0.402**	**0.002**	0.079	0.619	**0.469**	**0.002**	0.203	0.154	**0.45**	**0.002**	**0.322**	**0.031**
PCUN.L_NE	0.057	0.694	**0.367**	**0.023**	0.292	0.078	0.177	0.24	0.258	0.112	0.269	0.094
THA.R_NE	−0.105	0.453	**0.326**	**0.038**	0.095	0.564	0.161	0.274	0.077	0.632	0.267	0.084
MTG.R_NE	0.249	0.072	0.286	0.072	**0.538**	**0.005**	**0.338**	**0.018**	**0.467**	**0.002**	0.109	0.489
PreCG.R_NLp	**−0.396**	**0.005**	−0.383	0.061	**−0.515**	**0.001**	**−0.441**	**0.003**	**−0.457**	**0.004**	−0.306	0.06
IFGoperc.L_NLp	−0.181	0.202	−0.258	0.112	**−0.332**	**0.041**	−0.272	0.065	**−0.362**	**0.022**	**−0.4**	**0.009**
REC.L_NLp	0.102	0.393	0.015	0.926	0.071	0.663	0.042	0.774	0.589	0.577	−0.127	0.419
DCG.L_NLp	−0.15	0.292	−0.237	0.144	**−0.352**	**0.029**	**−0.365**	**0.011**	**−0.333**	**0.035**	−0.212	0.18
PCUN.L_NLp	−0.186	0.192	**−0.364**	**0.023**	**−0.375**	**0.021**	−0.269	0.07	**−0.352**	**0.027**	−0.308	0.051
MTG.R_NLp	**−0.431**	**0.001**	−0.256	0.109	**−0.552**	** <0.001**	**−0.48**	**0.004**	**−0.493**	**0.001**	−0.18	0.253

*P-values (<0.05) appeared in bold. ANG, angular gyrus; CUN, cuneus gyrus; DCG, median cingulate and paracingulate gyrus; Eg, global efficiency; IFGoperc, opercular part of inferior frontal gyrus; IFGtriang, triangular part of inferior frontal gyrus; IPL, inferior parietal; L, left; Lp, path length; MFG, middle frontal gyrus; MOG, middle occipital gyrus; MTG, middle temporal gyrus; NE, nodal efficiency; NLp, nodal path length; ORBmid, orbital part of middle frontal gyrus; ORBmid, orbital part of middle frontal gyrus; ORBsup, orbital part of superior frontal gyrus; PCUN, precuneus; PoCG, postcentral gyrus; PoCG, postcentral gyrus; PreCG, precentral gyrus; REC, rectus gyrus; R, right; SOG, superior occipital gyrus; THA, thalamus*.

### Mediation Path Between White Matter Hyperintensity and Cognitive Impairment

**Figure 3:**
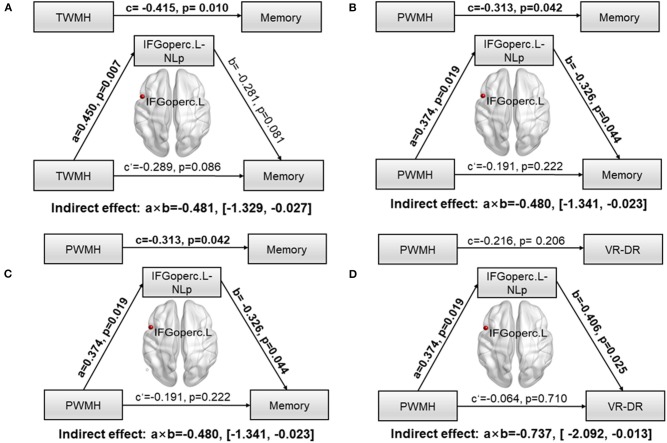
Path models of WMH on memory tested by the mediation analysis. Significant pathways were highlighted in bold characters. For each connection, the standard coefficient (a, b, c, c′) and *P*-value were shown. The indirect mediation effect (a × b) and its 95% confidence interval was also shown. **(A)** Association between TWMH and memory deficit was significantly mediated by NLp of IFGoperc.L (Indirect effect: −0.481; 95% confidence interval: −1.329, −0.027). **(B)** Association between PWMH and memory deficit was significantly mediated by NLp of IFGoperc.L (Indirect effect: −0.480; 95% confidence interval: −1.341, −0.023). **(C)** Association between TWMH and VR-DR was significantly mediated by NLp of IFGoperc.L (Indirect effect: −0.513; 95% confidence interval: −2.129, −0.019). **(D)** Association between PWMH and VR-DR was significantly mediated by NLp of IFGoperc.L (Indirect effect: −0.737; 95% confidence interval: −2.092, −0.013). WMH, white matter hyperintensity; IFGoperc, opercular part of inferior frontal gyrus; L, left; NLp, nodal path length; PWMH, periventricular white matter hyperintensity; TWMH, total white matter hyperintensity; VR-DR, Visual Reproduction–Delay Recall.

To further explore whether network parameters could fully or partially bridge WMH damage and CI, mediation models were constructed among altered network nodes, WMH volumes, and cognitive performances. We found that NLp in IFGoperc.L significantly mediated the relationship between TWMH volumes and the memory (indirect effect: −0.481; 95% confidence interval: −1.329, 0.027; Figure 3A). When substituting TWMH volumes with PWMH volumes or DWMH volumes in the mediation modal, we found just the pathway of PWMH achieved significance (indirect effect: −0.486; 95% confidence interval: −1.341, −0.023; Figure 3B), suggesting that the effect of WMH on memory was dominated by PWMH but not DWMH. Similarly, in order to address the exact mediation effect of IFGoperc.L on memory (visual memory or verbal memory), we substituted memory with the Z-scores of VR-DR or AVLT-DDR. This analysis showed that visual memory was the main memory function affected by IFGoperc.L and WMH (indirect effect: −0.736; 95% confidence interval: −2.092, −0.013; Figures 3C,D). Aside from significant mediation on NLp of IFGoperc.L, we found no other significant mediations.

## Discussion

In the present study, we used DTI tractography and graph theoretical analysis to calculate the topological properties of brain white matter network in subjects of WMH with or without CI and compared these parameters with those of healthy controls. We found that both WMH individuals showed decreased Eg and increased Lp at the level of whole brain compared with controls. WMH-CI group displayed more profound impairments of nodal Eg and NLp especially in PreCG.R, DCG.L, and MTG.R regions. The disrupted network properties were associated with CI and WMH burdens in the WMH-CI group. Furthermore, the study initially reported that NLp in IFGoperc.L fully or partially mediated the association between PWMH burdens and memory deficits, which may provide a comprehensive understanding on the development of WMH-related CI. These cross-sectional results detailed network disruptions in WMH-CI individuals and provided support for network measures as a disease marker for early WMH-CI diagnosis.

In agreement with previous literatures in healthy aging and CSVD (10, 31), our study in WMH population also confirmed that WMH volumes were associated with reduced network strength, Eg, and increased characteristic Lp. However, we did not detect significant discrepancies between WMH-no-CI group and WMH-CI group at global network level. One plausible explanation is that the degree of cognitive decline in this cohort is not serious. A total of 92.7% WMH-CI patients were diagnosed as mild CI (MCI) and 7.3% patients were diagnosed to have vascular dementia. Nevertheless, studies on WMH with MCI may be more helpful for us to find out the underlying mechanism why some WMH patients develop CI.

Next, we investigated nodal network properties among the three groups. Significant differences of Lp in six brain nodes and nodal efficiency in 19 brain nodes are distributed throughout the brain in subjects with WMH and CI. To briefly summarize, the altered nodes generally belong to default mode network [MFG.R, left medial orbitofrontal gyrus (ORBmid.L), ORBmid.R, ANG.R, PCUN.L, MTG.R] and frontoparietal network [left superior orbitofrontal gyrus (ORBsup.L), MFG.R, ORBmid.L, ORBmid.R, IFGoperc.L, PoCG.L, PoCG.R, IPL.R, ANG.R]. The full name and location of each node were displayed in Figure 2. It is widely accepted that default mode network and frontoparietal network are in charge of human memory retrieval (32, 33) and executive control functions (34, 35), respectively. Furthermore, decreased nodal efficiency in visual cortices (DCG.L; CUN.R; MOG.L) and thalamus (THA.R) could be crucial for visuospatial processing and executive functions (36). It is worth noting that disrupted NLp and nodal efficiency in DCG.L and MTG.R differed between WMH-no-CI and WMH-CI groups, indicating that these nodes may strongly be related to the early development of CI from WMH. Generally, most of the altered nodal network properties were located in the frontoparietal network in terms of spatial anatomic location, which matched with our previously functional study showing that functional connectivity was altered between the superior parietal gyrus and frontal regions in WMH-CI individuals (37). Therefore, we concluded that altered NLp and nodal efficiency may covalently participate in the development of WMH related-CI.

Information transfer and interaction between interconnected brain regions are believed to be a basis of the human cognitive processes (30, 38, 39). Eg and Lp represent the capacity for parallel processing from distributed brain regions; the longer the Lp, the lower the Eg (30). The significantly decreased Eg and increased Lp suggested potential damage to long contact fibers resulting from WMH, which in turn disrupted the integration of information communication between crucial neural networks and resulted in varying degrees of CI in multiple domains. In the present study, both network Eg and Lp were associated with CI in multiple domains. NLp and nodal efficiency are considered to be good parameters quantifying the efficiency of parallel information transfer among nodes in the network (30). Previous studies by resting-state MRI have demonstrated that disrupted nodal topological properties are strongly correlated with WMH related CI (40, 41). In terms of the white matter network, WMH can disrupt the integrity of white matter fibers and damage structural connections (42, 43), resulting in disrupted topological properties of nodes connected by the white fibers. Our study indicated that altered NLp and nodal efficiency in most regions are associated with WMH location and CI. Therefore, we further conduct a mediation analysis to explore whether altered NLp and nodal efficiency were involved in the relationship between WMH location and CI. We found only NLp in IFGoperc.L significantly mediated in PWMH-related memory deficit rather than DWMH. A recent study suggested that WMH can disrupt the left inferior fronto-occipital fasciculus which is one of the connections of the left inferior frontal gyrus (43). PWMH is more likely to disrupt white matter microstructure than DWMH. A previous study showed that PWMH has lower FA and more heterogeneous microstructure than DWMH (44), which is also consistent with the finding that PWMH can lead to CI in more domains than DWMH in the present study. Left inferior frontal gyrus plays a crucial role in maintaining normal memory. A previous study suggested that left inferior frontal gyrus activity was increased under conditions of high interference as compared to a low-interference condition of the same working task (45). Emerging literatures reported that left inferior frontal gyrus was associated with the ability to resolve interference efficiently during memory processes (46). A recent study showed that increasing activity in the inferior frontal gyrus may be involved in compensatory mechanisms to maintain working memory (47). Therefore, we concluded that PWMH may affect work memory through disrupting NLp in the IFGoperc.L. Additionally, in Stephan′s study, tract-specific integrity is associated with specific lobar gray matter volume, WMH, and CI. Performance on the test of memory is mostly associated with lobar gray volume. Frontal gray matter volume was found to be associated with FA of parahippocampal part of the cingulum, forceps major, and forceps minor. Inferior fronto-occipital fasciculus and inferior longitudinal fasciculus connecting the inferior frontal gyrus were prone to WMH occurrence (12). In the present study, we found that PWMH leads to memory deficit through affecting nodal properties of the IFGoperc.L. The left inferior frontal gyrus plays a crucial role in maintaining normal memory. Maybe further studies could be performed to evaluate the associations among regional frontal gray matter volume, white matter integrity, and CI.

This is an initial cross-sectional study investigating the sole effects of WMH on CI at the level of network and node using DTI tractography and graph theoretical analysis. Some limitations should be addressed. Firstly, the distinctions between deep and periventricular WMH may be debatable (48). Both extent and spatial location of WMH associated with increasing occurrence of CI (49). More accurate quantitative method of regional WMH should be applied in future work. Secondly, although deterministic tractography is a classical method widely used to analyze white matter fibers (15, 24), it is unable to track crossed or twisted fibers and may lead to measurement bias (50, 51). We will consider to perform fiber tracking using more advanced data reconstruction method, e.g., probabilistic tractography which may be more feasible as it can overcome fiber crossings and is robust to image noise (52). Thirdly, the parcellation of the brain regions might influence the network properties. Although AAL-90 has been used in other network-based studies in CSVD (15, 23–25), it is a relative labeling technique that consists of unequal-sized brain regions to parcel the brain regions for network construction. Because volume normalization method may potentially over- or under-compensate for volume-driven effect on the fibers and result in new confounding factors (53). We did not normalize the volume sizes of the regions. For future work, a more modern and accepted labeling scheme should be used at least for gray matter parcellations, e.g., https://mindboggle.readthedocs.io/en/latest/labels.html. Finally, the sample size in this study is relatively small and the nature of this study is cross-sectional; no causal inferences or directionality can be made. We are continuing to recruit new participants and follow them up to validate our findings. Overall, longitudinal and large-sample studies are required to further confirm these findings, and an individualized evaluation system for disease progression in WMH patients should be formulated ultimately in the future.

## Conclusions

In conclusion, brain structural network in WMH-CI is significantly disturbed, and this disturbance is related to the severity of WMH and CI. Increased NLp of IFGoperc.L was shown to be a mediation framework between PWMH and WMH-related memory deficit. Taking white matter network analysis into consideration may be beneficial for early diagnosis of WMH-related CI and understanding the underlying mechanism of CI caused by WMH.

## Data Availability Statement

The datasets generated for this study are available on request to the corresponding author.

## Ethics Statement

The studies involving human participants were reviewed and approved by the Ethics Committee of Nanjing Drum Tower Hospital. The patients/participants provided their written informed consent to participate in this study. Written informed consent was obtained from the individual(s), and minor(s)' legal guardian/next of kin, for the publication of any potentially identifiable images or data included in this article.

## Author Contributions

All authors listed have made a substantial, direct and intellectual contribution to the work, and approved it for publication.

## Conflict of Interest

The authors declare that the research was conducted in the absence of any commercial or financial relationships that could be construed as a potential conflict of interest.

## Abbreviations

AAL: automated anatomical labeling
AD: Alzheimer's disease
ANCOVA: analysis of covariance
ANG: angular gyrus
ANOVA: analysis of variance
AVLT-DDR: auditory verbal learning test–long delayed recall
BNT: Boston Naming Test
CDT: clock drawing test
CI: cognitive impairment
CSVD: cerebral small vessel disease
CUN: cuneus
CVF: category language fluency
Cp: clustering coefficient
DCG: median cingulate and paracingulate gyri
DTI: diffusion tensor imaging
DWMH: deep white matter hyperintensity
Eg: global efficiency
Eloc: local efficiency
FA: fractional anisotropy
FDR: false discovery rate
FLAIR: fluid-attenuated inversion recovery
FN: fiber number
HAMA: Hamilton Anxiety Rating Scale
HAMD: Hamilton Depression Rating Scale
HC: healthy controls
IFGoperc: opercular part of inferior frontal gyrus
IFGtriang: triangular part of inferior frontal gyrus
IPL: inferior parietal
L: left
Lp: path length
MFG: middle frontal gyrus
MCI: mild cognitive impairment
MMSE: Mini Mental State Examination
MoCA-BJ: Beijing version of the Montreal Cognitive Assessment
MOG: middle occipital gyrus
MTG: middle temporal gyrus
NE: nodal efficiency
NLp: nodal path length
ORBmid: orbital part of middle frontal gyrus
ORBmid: orbital part of middle frontal gyrus
ORBsup: orbital part of superior frontal gyrus
PCUN: precuneus
PoCG: postcentral gyrus
PoCG: postcentral gyrus
PreCG: precentral gyrus
PWMH: periventricular white matter hyperintensity
R: right
REC: gyrus rectus
SOG: superior occipital gyrus
Stroop A: Stroop Color and Word Test-A
Stroop B: Stroop Color and Word Test-B
Stroop C: Stroop Color and Word Test-C
TBV: total brain volume
THA: thalamus
TMT-A: Trail Making Test-A
TMT-B: Trail Making Test-B
TWMH: total white matter hyperintensity
VR-C: visual reproduction-copy
VR-DR: visual reproduction-delay recall
WMH: white matter hyperintensity.
